# Risk stratification for febrile neutropenia in patients with testicular germ cell tumors

**DOI:** 10.1002/cam4.1317

**Published:** 2018-01-19

**Authors:** Angelika Terbuch, Florian Posch, Richard Partl, Brigitte Zurl, Thomas Bauernhofer, Martin Pichler, Joanna Szkandera, Georg C. Hutterer, Karl Pummer, Karin S. Kapp, Herbert Stöger, Armin Gerger, Michael Stotz

**Affiliations:** ^1^ Division of Oncology Department of Internal Medicine Comprehensive Cancer Center Graz Medical University of Graz Graz Austria; ^2^ Research Unit Genetic Epidemiology and Pharmacogenetics Medical University of Graz Graz Austria; ^3^ Department of Therapeutic Radiology and Oncology Comprehensive Cancer Center Graz Medical University of Graz Graz Austria; ^4^ Center for Biomarker Research in Medicine (CBmed) Graz Austria; ^5^ Department of Experimental Therapeutics The University of Texas MD Anderson Cancer Center Houston Texas; ^6^ Department of Urology Medical University of Graz Graz Austria

**Keywords:** testicular cancer, febrile neutropenia, risk factors, radiotherapy, chemotherapy

## Abstract

The aim of this study was to detect risk factors for febrile neutropenia (FN) in patients with testicular germ cell tumors (TGCT). In this retrospective cohort study at the Medical University of Graz, we included 413 consecutive TGCT patients who received adjuvant or curative treatment with cisplatin‐based chemotherapy. FN occurred in 70 (16.9%) of 413 patients. In univariable logistic regression, higher age (odds ratio (OR) per 5 years = 1.17, 95% CI: 1.02–1.35, *P* = 0.022), reduced performance status (PS) (OR = 2.73, 1.47–5.06, *P* = 0.001), seminomatous histology (OR = 2.19, 1.26–3.78, *P* = 0.005), poor IGCCCG risk class (OR = 4.20, 1.71–10.33, *P* = 0.002), and prior radiotherapy (pRTX) (OR = 8.98, 2.09–38.61, *P* = 0.003) were associated with a higher risk of FN. In multivariable analysis adjusting for age and risk classification, only poor PS (OR = 2.06, 1.05–4.03, *P* = 0.035), seminomatous histology (OR = 2.08, 1.01–4.26, *P* = 0.047), and pRTX (OR = 7.31, 1.61–33.17, *P* = 0.010) prevailed. In the subgroup of seminoma patients (*n* = 104), only pRTX predicted for FN risk (OR = 5.60, 1.24–25.34, *P* = 0.025). Five of eight seminoma patients with pRTX developed FN (63%), as compared to 22 FN cases (23%) in the 96 seminoma patients without pRTX (*P* = 0.027). The eight seminoma patients who received pRTX had significantly lower pre‐chemo white blood counts (4.7 vs. 6.5 G/L), neutrophil counts (3.2 vs. 4.3 G/L), and platelet counts (185 vs. 272 G/L) than patients without pRTX (all *P* < 0.0001). TGCT patients with a reduced performance status or who had been previously treated with radiotherapy have an increased risk for neutropenic fever during chemotherapy.

## Introduction

Testicular germ cell tumors (TGCT) represent the most common solid malignancy among men aged 15–40 years and can be divided into two subgroups: seminomatous (SGCT) and nonseminomatous germ cell tumors (NSGCT) 1. Long‐term prognosis of testicular germ cell tumors (TGCT) is excellent. The 5‐year survival rate of patients with TGCT in stage I approximates 99%. Treatment options for stage I seminoma are active surveillance, adjuvant treatment with one cycle of carboplatin or adjuvant radiotherapy 2, 3. NSGCT patients with stage I disease can be managed with active surveillance or may be offered one adjuvant chemotherapy cycle with bleomycin, etoposide, and cisplatin (BEP) 4. For metastatic cases, the International Germ Cell Cancer Collaborative Group (IGCCCG) has identified three prognostic groups: good, intermediate, and poor risk. With optimal management the 5‐year survival rate approximates 91%, 79% and 48% in metastatic disease with good, intermediate and poor prognosis 5, 6. Depending on the prognostic group three or four cycles of BEP represent the standard of care for relapsed or metastatic disease. In stage II A disease, also radiotherapy for seminoma and retroperitoneal lymph node dissection (RPLND) for nonseminoma are valid treatment options 1, 5.

However, treatment is associated with non‐negligible complications and a negative impact on quality of life 7, 8, 9, 10, 11. Therefore, optimizing the complication rate of TCGT treatment holds promise to further increase the already favorable prognosis of these patients.

Febrile neutropenia (FN) is a serious complication of myelosuppressive chemotherapy. FN rates during curative BEP chemotherapy are between 10 and 20%. Routine prophylactic use of granulocyte colony‐stimulating factors (G‐CSF) is not recommended 12. A personalized FN risk stratification approach to the patient with TCGT in need of systemic chemotherapy may optimize the indication and thus risk‐benefit ratio of prophylactic G‐CSF support in this population. Thus, the aim of this study was to identify prognostic factors for FN in order to delineate subgroups with the highest risk of FN.

## Materials and Methods

### Subjects

All consecutive patients (*n* = 960) with histologically confirmed TGCT, presenting to the Division of Oncology at the Medical University of Graz between January 1994 and September 2013, were retrospectively reviewed. Patients were initially staged using computed tomographic (CT) scans of the abdomen, CT scan or X‐ray of the chest, and postoperative tumor markers α‐fetoprotein (AFP), human chorionic gonadotropin (HCG), and lactate dehydrogenase (LDH). Patients with disseminated disease were stratified according to the IGCCCG risk classification 13, 14.

Follow‐up data were retrieved until January 2015. Follow‐up investigations at our center were performed according to a local protocol and were adapted in 2007 and 2012 according to recent publications 15, 16, 17. Because the primary endpoint of this study was the risk of febrile neutropenia in patients undergoing cisplatin‐based chemotherapy, patients who were managed with active surveillance, adjuvant or curative radiotherapy, adjuvant carboplatin and did not experience relapse were excluded from further analysis (*n* = 547). A total of 413 patients who received adjuvant or curative cisplatin‐based chemotherapy during the course of disease were selected for this study (Table 1). A total of 377 (91%) of 413 patients had not received any treatment (except orchiectomy) prior to cisplatin‐based chemotherapy, eight (2%) patients had undergone adjuvant or curative radiotherapy to paraaortic/iliac lymph nodes prior to chemotherapy and one (0%) patient had received one cycle of adjuvant carboplatin. Because 27 patients received several cycles of cisplatin‐based chemotherapy, all cycles were counted until an episode of FN occurred. In patients without any FN episode, all cycles of cisplatin‐based chemotherapy were counted. Electronic and paper medical records of all 413 consecutive TGCT patients were retrospectively reviewed, and febrile neutropenia was documented in our in‐house administrative system. FN was defined as an oral temperature of >38.3°C or two consecutive readings of >38.0°C for 2 h and an absolute neutrophil count (ANC) of <0.5 × 10^9^/L, or expected to fall below 0.5 × 10^9^/L 18.

**Table 1:** Baseline characteristics of the patient population – Distribution overall and by febrile neutropenia

Variable	*n* (% missing)	Overall (*n* = 413)	No febrile neutropenia during CTX (*n* = 343)	Febrile neutropenia during CTX (*n* = 70)	*P* a
Demographic characteristics					
Age	413 (0.0%)	34 [27–40]	33 [27–40]	36 [28–42]	0.052
BMI	274 (34%)	25 [22–27]	25 [22–27]	24 [22–26]	0.439
Smoker or ex‐smoker	298 (28%)	169 (57%)	146 (59%)	23 (47%)	0.131
Karnofsky index < 100%	371 (10%)	61 (16%)	41 (13%)	20 (30%)	0.001
Clinicopathological variables					
Seminomatous histology	408 (0%)^b^	104 (25%)	77 (23%)	27 (39%)	0.004
T Stage	375 (9%)	/	/	/	0.088
pT1	/	164 (44%)	142 (45%)	22 (36%)	/
pT2	/	143 (38%)	121 (39%)	22 (36.0%)	/
pT3	/	68 (18%)	51 (16%)	17 (28%)	/
pT4		0 (0%)	0 (00.0%)	0 (0.0%)	
Adjuvant treatment^c^	413 (0%)	141 (34%)	126 (37%)	15 (21%)	0.014
IGCCCG	272 (0%)				
Good risk		203 (75%)	167 (77%)	36 (65%)	0.112
Intermediate risk		36 (13%)	28 (13%)	8 (15%)	
Poor risk		33 (12%)	22(10%)	11 (20%)	
Synchronous metastasis	272 (0%)	232 (85%)	185 (85%)	47 (85%)	0.970
Primary G‐CSF support	345 (16%)	60 (17%)	48 (17%)	12 (19%)	0.751
Prior treatment^d^	413 (0%)				0.001
No prior treatment		377 (91%)	318 (93%)	59 (84%)	
Radiotherapy		8 (2%)	3 (1%)	5 (7%)	
Adjuvant carboplatin		1 (0%)	1 (0%)	0 (0%)	
2 cycles PEB		2 (0%)	1 (0%)	1 (1%)	
3 cycles PEB		2 (0%)	1 (0%)	1 (1%)	
4 cycle PEB		5 (1%)	2 (1%)	3 (4%)	
Multiple CTX schemes		18 (4%)	17 (5%)	1 (1%)	
Current treatment^e^	413 (0%)				
PEB		395 (96%)	335 (98%)	60 (86%)	0.0001
PE		5 (1%)	3 (1%)	2 (3%)	
PEI		11 (3%)	5 (1%)	6 (9%)	
TIP		1 (0%)	0 (0%)	1 (1%)	
VIDE		1 (0%)	0 (0%)	1 (1%)	
Laboratory parameters (pre CTX)					
Leukocytes (G/L)	333 (19%)	7.0 [5.6–8.8]	7.4.6 1 [5.7‐8.8]	7.0 [5.5–8.6]	0.699
Neutrophiles (G/L)	317 (23%)	4.6 [3.4–6.9]	4.6 [3.4–6.9]	4.9 [3.4–6.0]	0.633
Lymphocytes	318	1.6 [1.2–2.0]	1.6 [1.2–2.0]	1.3 [0.9–1.9]	0.038
Thrombocytes (G/L)	332 (20%)	254.0 [213.0–313.0]	252.0 [213–310.0]	273.0 [207.0–316.0]	0.452
Hemoglobin (g/dl)	338 (18%)	15.3 [14.2–16.1]	15.3 [14.5–16.1]	14.6 [13.1–15.6]	0.001
AFP	348 (16%)	4.6 [2.5–38.0]	4.6.0 [2.5–35.3]	4.2 [2.4–48.2]	0.880
ß‐HCG	332 (20%)	1.2 [1.2–14.8]	1.2 [1.2–14.9]	1.2 [1.2–14.8]	0.335
LDH	334 (19%)	192.0 [156.0‐285.0]	188.0 [155.0–264.0]	235.0 [170.0–477.0]	0.005

Continuous data are reported as medians with 25th percentile–75th percentile in the squared brackets, and categorical data are reported as absolute frequencies and (percentages) in parentheses. Percentages are calculated by referring only to the patients without missing values (i.e., not to the total number of patients if missing values are present).

FN, febrile neutropenia; BMI, body mass index; TGCT, testicular germ cell tumor; IGCCCG, International Germ Cell Cancer Collaborative Group; AFP, alpha fetoprotein; ß‐HCG, beta human choriogonadotropin; LDH, Lactate dehydrogenase.

a p represents test for difference between FN and No FN.

b a distinction between seminoma and nonseminoma could be made in 408 cases (in the other five cases no distinction was possible due to necrosis).

c FN occurring during adjuvant treatment.

d Prior treatment includes all given treatment before cisplatin‐based chemotherapy; in case of several cycles of cisplatin‐based chemotherapy, all cycles were counted until FN occurred; in patients without any FN episode, all cycles of cisplatin‐based chemotherapy were counted.

e Current treatment means chemotherapy regimen under which FN occurred; in patients without any FN episode, last chemotherapy regimen is cited.

The study was approved by the Institutional Review Board of the Medical University of Graz (No. 26‐196 ex 13/1).

### Statistical methods

All statistical analyses were performed using Stata (Version 14.0, Stata Corp., Houston, TX) and IBM SPSS Statistics (Release 23.0.0. 2015. Chicago (IL), USA: SPSS Inc., an IBM Company). Continuous variables were reported as medians [25th–75th percentile] and count data as absolute frequencies (%). Means between two or more groups were compared with t‐tests, rank‐sum tests, and Kruskal–Wallis tests, respectively. Spearman's rank‐based correlation coefficient was used for examining the correlation between radiotherapy parameters and blood counts. The association between FN and clinical covariables was quantified with uni‐ and multivariable logistic regression 19.

## Results

### Analysis at Baseline

#### Characterization of FN episodes

During a total number of 1.196 chemotherapy cycles [median: 3, (IQR: 2–3, range: 1–6)], we observed 70 episodes of febrile neutropenia (16.9%) (Table 1). In 55 (79%) of these 70 events, the episode occurred during the first cycle of treatment. In 10 (14%), two (3%), and three (4%) of patients, the episode occurred during the 2nd, 3rd and 4th cycle of treatment. The median time between CTX start and FN onset was 14 days (IQR: 12–15, range: 7–18). One (1%) FN episode was fatal, and 56 patients (80%) had to be hospitalized. The median time in hospital was 7 days (IQR: 6–8, range: 3–12), and the median number of days with an absolute neutrophil count below 0.5 G/L was 2 (IQR: 2–3, range: 1–7). Sixty (14.5%) of 413 patients received primary GCSF support. Twelve (19%) FN episodes occurred despite primary GCSF support. There was very little treatment delay (median: 0 days (IQR: 0–0, range: 0–7). Three patients (4%) developed a second FN episode.

#### Prediction of FN

In univariable logistic regression, higher age (odds ratio (OR) per 5 years = 1.17, 95% CI: 1.02–1.35, *P* = 0.022), reduced performance status (PS) (OR = 2.73, 1.47–5.06, *P* = 0.001), seminomatous histology (OR = 2.19, 1.26–3.78, *P* = 0.005), poor IGCCCG risk class (OR = 4.20, 1.71–10.33, *P* = 0.002), and prior RTX (pRTX, OR = 8.98, 2.09–38.61, *P* = 0.003) were associated with a higher risk of FN. In multivariable analysis adjusting for age and risk classification, only poor PS (OR = 2.06, 1.05–4.03, *P* = 0.035), seminomatous histology (OR = 2.08, 1.01–4.26, *P* = 0.047), and pRTX (OR = 7.31, 1.61–33.17, *P* = 0.010) prevailed (Table 2). In the subgroup of seminoma patients (*n* = 104), only pRTX predicted for FN risk (OR = 5.60, 1.24–25.34, *P* = 0.025). In detail, the eight seminoma patients who received pRTX had significantly lower pre‐chemo white blood counts (4.7 vs. 6.5 G/L), neutrophil counts (3.2 vs. 4.3 G/L), and platelet counts (185 vs. 272 G/L) than patients without pRTX (all *P* < 0.0001). Five of eight seminoma patients with pRTX developed FN (63%), as compared to 22 FN cases (23%) in the 96 seminoma patients without pRTX (*P* = 0.027). We did not observe a significant correlation between white blood and platelet count and certain radiation parameters like radiation dose, radiation field size, and mean irradiated bone volume receiving at least 10 Gy (V10), 20 Gy (V20), and 25 (Gy) (data not shown).

**Table 2:** Predictors of FN in TGCT patients undergoing CTX – uni‐ and multivariable logistic regression

Variable	Univariable OR	95% CI	*P*	Multivariable OR adjusted for age and risk classification	95% CI	*P*
Demographic characteristics						
Age (per 5 years increase)	1.17	1.02–1.35	0.022	N/A	N/A	N/A
BMI (for 5 kg/m² increase)	0.90	0.65–1.25	0.537	0.83	0.58–1.19	0.302
Smoker or ex‐smoker	0.62	0.34–1.15	0.133	0.66	0.35–1.23	0.191
Karnofsky index < 100%	2.73	1.47–5.06	0.001	2.06	1.05–4.03	0.035
Clinicopathological variables						
Seminomatous histology	2.19	1.26–3.78	0.005	2.08	1.01–4.26	0.047
T Stage						
pT1	Ref	Ref	Ref			
pT2	1.17	0.62–2.22	0.623	1.21	0.63–2.34	0.562
pT3	2.15	1.06–4.37	0.034	1.83	0.88–3.83	0.106
IGCCCG						
Adjuvant chemotherapy (Reference)	Ref	Ref	Ref			
good risk	1.81	0.95–3.45	0.071	N/A	N/A	N/A
Intermediate risk	2.40	0.93–6.21	0.071	N/A	N/A	N/A
Poor risk	4.20	1.71–10.33	0.002	N/A	N/A	N/A
Synchronous metastasis	1.02	0.44–2.35	0.970	0.90	0.38–2.16	0.819
Primary GCSF support	1.12	0.56–2.26	0.751	0.81	0.40–1.71	0.572
Prior Treatment^a^						
No prior treatment	Ref	Ref	Ref			
PEB chemotherapy	6.74	1.76–25.83	0.005	5.50	1.37–22.11	0.016
Other chemotherapy	0.30	0.04–2.29	0.245	0.22	0.03–1.72	0.149
Radiotherapy	8.98	2.09–38.61	0.003	7.31	1.61–33.17	0.010
Current chemotherapy^b^ (PEB = reference)						
Non PEB chemotherapy	6.98	2.65–18.40	<0.0001	4.74	1.72–13.08	0.003
Laboratory parameters (pre Chemotherapy)						
Leukocytes (per 1G/L increase)^c^	0.97	0.89–1.07	0.560	0.96	0.86–1.06	0.395
Neutrophils (per 1G/L increase)	1.03	0.91–1.16	0.613	1.00	0.88–1.14	0.949
Lymphocytes (per 1G/L increase)	1.07	0.87–1.31	0.550	1.10	0.89–1.37	0.371
Thrombocytes (per 100G/L increase)	1.04	0.76–1.43	0.801	0.91	0.65–1.26	0.561
Hemoglobin (per 1 g/dl)	0.78	0.67–0.91	0.001	0.82	0.69–0.97	0.022
AFP per doubling	1.04	0.95–1.12	0.398	0.99	0.90–1.09	0.887
ß‐HCG per doubling	1.04	0.98–1.10	0.242	1.01	0.94–1.08	0.848
Preoperative LDH per doubling	1.45	1.10–1.91	0.009	1.25	0.87–1.81	0.229

FN, febrile neutropenia; TGCT, testicular germ cell tumor; BMI, body mass index; N/A, not applicable; IGCCCG, International Germ Cell Cancer ollaborative Group; AFP, alpha fetoprotein; ß‐HCG, beta human choriogonadotropin; LDH, lactate dehydrogenase.

a Prior treatment includes all given treatment before FN occurred.

b Current treatment means chemotherapy regimen under which FN occurred.

c One patient with extreme Leukocyte count excluded.

## Discussion

The present study demonstrated and confirmed that FN is a frequent complication in TGCT patients undergoing curative or adjuvant cisplatin‐based chemotherapy. In detail, the FN risk in our cohort was 17%. However, treatment delay was negligible and the case fatality rate of FN in TGCT appears to be very low. Nevertheless, 80% of patients with FN had to be admitted to hospital for a median duration of 1 week, which obviously comprises quality of life during the course of chemotherapy and increases healthcare costs. With establishment of current guidelines and identification of “low‐risk” patients, the admission rate nowadays might be lower. However, primary G‐CSF support may have a role in reducing the burden of FN in this population. Guidelines of the major societies in the field, such as the EORTC algorithm, recommend primary G‐CSF support when the predicted FN risk is above 20%. In case the FN risk is assumed to be between 10% and 20%, the decision on whether or not to prescribe primary G‐CSF support should be supported by an individual assessment of FN risk factors for each patient 12. Our data show that with a FN risk of 17%, TCGT patients fall into this “individual risk assessment” group. However, the risk factors that should be considered for this individual risk assessment according to the EORTC algorithm, such as age > 65 years poor performance status, poor nutrition status, female sex, anemia, and comorbidity, are not very prevalent in the often young and minimally comorbid TGCT population. Therefore, our study aimed to identify FN risk factors specifically for TGCT patients in order to facilitate FN risk assessment.

In detail, we could confirm a previous report by Feldman et al. that even age above 50 years is an important risk factor for FN in TGCT patients. These authors observed a 44% FN risk in TCGT patients above 50 years of age, which prompted them to recommended primary G‐CSF administration for all patients in this age group 20. Two other strong FN risk factors identified by our analysis were poor performance status and poor IGCCCG risk classification.

Moreover, seminomatous histology and prior radiotherapy emerged as risk factors for FN in the overall study cohort. Because radiotherapy is only used as adjuvant or curative treatment in SGCT and not NSGCT patients, this might explain why seminomatous histology appeared to be a risk factor for developing FN (“confounding by radiotherapy”). Clinically, a link between prior radiotherapy and a higher FN risk is highly plausible 21. The acute depletion of bone marrow components following irradiation has been ascribed to the direct effect of radiation depleting the stem cell compartment 22. The ability of the bone marrow compartment to recover and regenerate is dependent on the volume of bone marrow within the irradiated field. Radiotherapy to paraaortal lymph nodes involves around 25% of bone marrow 22, 23. In our analysis, the eight seminoma patients who received pRTX had significantly lower pre‐chemo white blood counts, neutrophil counts, and platelet counts than patients without pRTX (all *P* < 0.0001). The mean time from radiotherapy to relapse and subsequent chemotherapy was 1 year. This supports the hypothesis that irradiated bone marrow might not have the ability to recover or that a time interval of 1 year is too short for recovery which makes the risk of FN for relapsed seminoma patients that high.

We did not observe that the magnitude of reduction in mean blood count was associated with certain radiation parameters like radiation dose or radiation field size among seminoma patients even though there were differences in radiation dose and volume. The differences in dose and volume prescriptions are attributable to the long observation period and the change in radiation dose from 30 Gy to 20 Gy after the results from the EORTC trial 30942 in 2005 24, 25.

Our study is limited by its retrospective character and the small sample size of the subgroup of relapsed irradiated seminoma patients. The most used regimen in our study was PEB chemotherapy (in 96% of patients) and only 4% received multiple chemotherapy regimens. Our risk stratification therefore mainly applies for TGCT patients who are treated with PEB chemotherapy as first‐line treatment.

In summary, we observed a considerable risk of FN in men with TGCT undergoing cisplatin‐based adjuvant or curative chemotherapy. Our study identified (1) higher age, (2) poor performance status, (3) poor IGCCCG risk classification, and (4) prior radiotherapy in the seminoma subpopulation as risk factors for FN in patients with testicular cancer.

## Conflict of Interest

None of the contributing authors have any conflict of interests, including specific financial interests and relationships and affiliations relevant to the subject matter or materials discussed in the manuscript.
